# Reducing social security contribution rate on the financial performance of state-owned and non-state-owned manufacturing industries in the post-epidemic era

**DOI:** 10.1371/journal.pone.0287596

**Published:** 2023-06-23

**Authors:** Liangchen Zhang, Guangli Yang

**Affiliations:** School of Accountancy, Guangzhou Xinhua University, Guangzhou, China; University of Almeria: Universidad de Almeria, SPAIN

## Abstract

Social insurance is an essential component of a contemporary social security system since it protects people’s fundamental well-being, but it also incurs a heavy cost for businesses. If social security costs are excessively high, business profitability will suffer, and innovation will be discouraged. The most affected companies would be those in labor-intensive industries and medium-sized enterprises. Chinese businesses have suffered severe losses as a result of the COVID-19 outbreak. Given the circumstance, China enacted additional tax cuts and preferential social insurance premium plans. This article suggests a lower ratio of contribution as a strategy to cut the cost of social insurance premiums for businesses, given the growth of the social security fund in recent years and the proportion of participants to recipients in pension funds. It would be possible to increase firm profitability and lessen the impact of COVID-19 on industries by minimizing this operation burden. In order to compare the financial performance of state-owned manufacturers (SOMs) to that of their non-state-owned peers, who have a lower ratio of contribution, this study uses a multiple regression model. The ratio of contributions was inversely correlated with an enterprise’s financial performance. In other words, financial performance will improve as the ratio of contribution lowers; nevertheless, this effect is more pronounced in SOMs. The final section of this study proposed optimized approaches for social insurance premiums reform.

## 1. Introduction

Public insurance is an essential component of a contemporary social security system. Compared with developed economies, China’s social insurance system has a shorter history, with its beginning marked by the implementation of the Regulations of the People’s Republic of China on Labor Insurance in 1951, a milestone in China’s exploration of social insurance reform. 2011 saw the introduction of the Social Insurance Law of the People’s Republic of China, which was a remarkable event in the legalization of the social insurance system in China. Zhao et al. [1] argued that social insurance is committed to solving people’s livelihood problems, so social insurance in China is made up of five components: pension insurance, medical insurance, work injury insurance, unemployment insurance, and maternity insurance, which cover all aspects of residential lives. Li [2] pointed out that social insurance pursues fairness by redistributing national income to protect the rights and interests of general residents and building a well-off society in all aspects.

**Fig 1:**
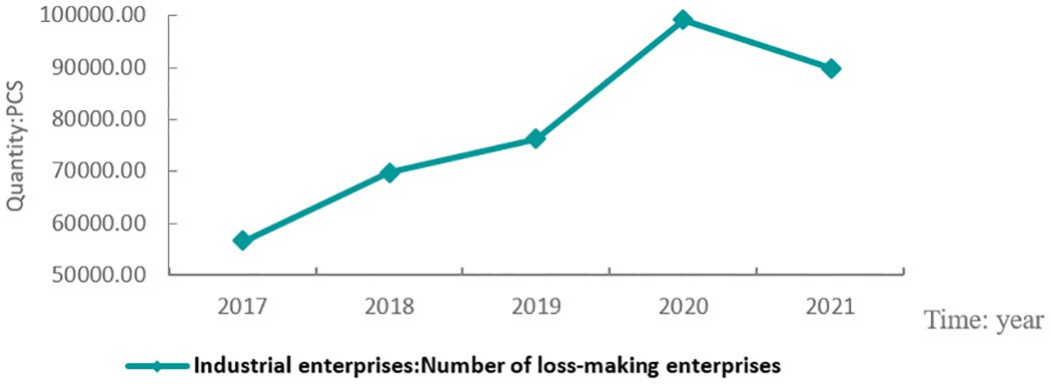
Number of industrial enterprises with losses, 2017–2021.

Li et al. [3] pointed out that despite its basic protection and welfare function, the social insurance is a kind of cost. Li [4] explained that enterprises and employees share the social insurance costs proportionately according to the statutory ratio. As a result, the payment of employee social insurance has been a cost for enterprises. He [5] argued that if the burden of social security contributions is excessively high, it will undermine the profitability of enterprises and discourage innovation. The most affected businesses would be those in labor-intensive industries and medium-sized enterprises. 2019 saw the introduction of the Comprehensive Plan for Reducing Social Insurance Premium Rates, and COVID-19 caused significant losses to enterprises. More than 90,000 companies suffered losses in 2020 (as shown in Fig 1). To mitigate the economic dilemma, China issued a number of preferential policies on social insurance premiums, such as deferred contribution policy for pensions and unemployment insurance, reflecting China’s determinant effort to reduce the burden of social insurance premiums for firms. China is committed to exploring a healthier and optimized social security system, so related research is needed. Uncertain economic outlook exposes Chinese economy and stability to risks, challenging Chinese mission of national revitalization. The real economy lays the foundation of the national economy, so an efficient and stable real economy is of utmost importance. To meet this end, it is necessary to adjust the ratio of contribution for social insurance premium according to the new social condition as a driving force to promote technological upgrade and innovation for real enterprises.

**Fig 2:**
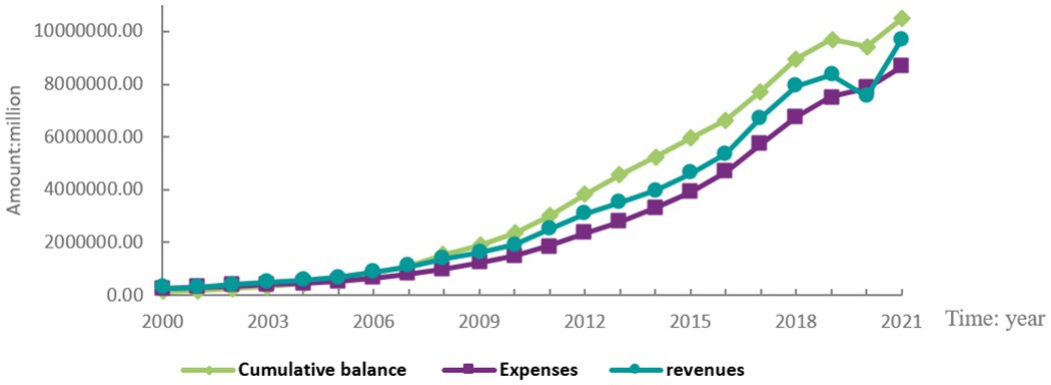
National social security fund income, expenditure, and accumulated balance.

**Fig 3:**
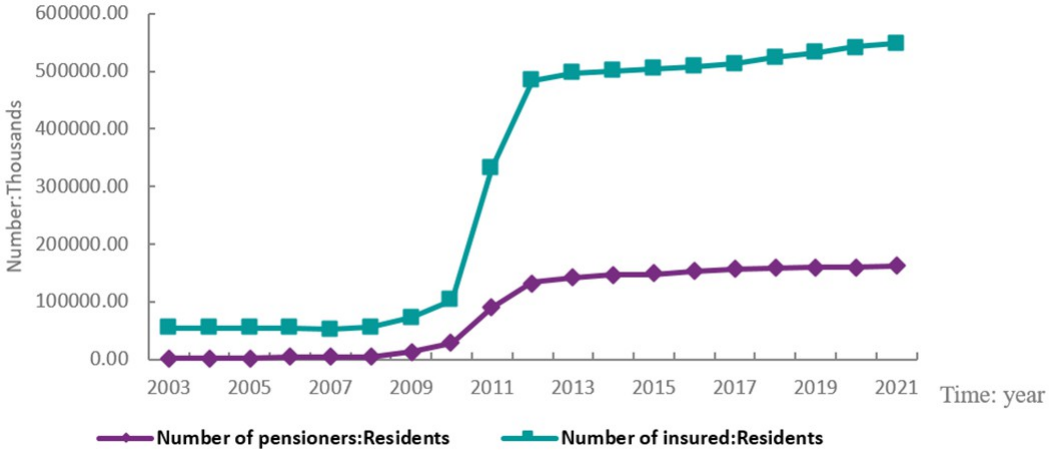
Number of recipients and participants of the national social security fund.

The National Bureau of Statistics reports that since 2000, the income, expenses, and accrued balance of China’s social security fund have all increased. This information is displayed in Fig 2. The income and the accrued balance of China’s social security fund decreased as a result of the nation’s enactment of more benevolent social security measures in 2020 to mitigate the impact of the pandemic. Since 2010, the social security fund has seen a sharp increase in participant size, far outpacing the number of fund beneficiaries (see Fig 3). The ratio of social insurance contributions was reduced without affecting beneficiaries’ benefits because the number of companies recording losses increased significantly during the COVID-19 pandemic in China. As a result, businesses’ operational expenses dropped while their profits rose, which was advantageous for business growth and efficiency.

Main contributions of this thesis is discovered that COVID-19 led to an increasing number of enterprises suffering losses in China, so a lower ratio of social security contributions without guaranteed benefits for the normal operation of social security funds could reduce the labor cost of enterprises, increase cash flow and profits, and restore the vitality of enterprise production. This thesis conducts an empirical study on the impact of a lower ratio of social security contributions on the financial performance of state-owned manufacturers (SOM) and non-state-owned ones, respectively. Research shows that a lower ratio of contribution would improve the financial performance, and such effects are more profound and apparent in SOMs. This thesis provides a reference for the recovery of SOMs and non-SOMs in the post-COVID era.

## 2. Theoretical framework

### 2.1 Theoretical review

China’s social insurance fund has three contributors: the employee, the employer, and the official authority. The ratios of contributions applied to employees and employers are different, while the remaining subsidies are attributable to the national budget. The ratio of contribution for enterprises is formulated in the national social insurance policy, affecting a company’s profitability. There are two major viewpoints about the impact of social insurance contributions: "incentive theory" and "cost theory".

Yuan and Luo [6] pointed out the "incentive theory" emphasizes the positive effect of social insurance on productivity. It believes that a higher ratio of contributions is one of the benefits for employees, motivating their creativity and innovation capabilities. Fully paid social insurance premiums could be an incentive for employees as their livelihood is secured, which in turn makes them more confident in their job and enhances stability. Motivated employees would have greater potential for creation. Meanwhile, companies that fully pay social insurance for their employees are also more competitive in talent recruitment. At the same time, to reduce the burden of social insurance payments, companies will also improve their investment to optimize staff structure and productivity, thus improving their financial performance.

Zhang et al. [7] pointed out that the “cost theory” focuses on the fact that social insurance is a major cost by nature, and when the ratio of statuary social insurance contribution is excessively high, it will squeeze investment in innovation and R&D, let alone the development of productivity. The biggest victim of heavy social security contributions is the labor-intensive industry. To get rid of such a heavy burden, companies may refuse or underpay social security for employees. As the total labor cost is fixed, a higher contribution to social insurance means less disposable income received by the employee, who would be less motivated to work when their expectations were not met. Salary expenses have been the main cost of labor-intensive manufacturers, so the "cost theory" is the appropriate basis for the study.

### 2.2 Literature review

Foreign scholar Griliches [8] found through empirical research that a company’s R&D (research and development) investment is positively correlated with the industrial added-value. By contrast, the social insurance for employees represents the welfare provided by employers. Therefore, to attract top talents of R&D and innovation, a salary package with higher social insurance premiums could be one of the most effective incentives to motivate and stimulate employees’ working enthusiasm, which would further enhance the enterprises’ competitiveness and productivity [9]. Wu et al. [10], Shen et al. [11], and Ma and Tian [12] argued that social security might have a substitution effect on production factors, as some enterprises may redistribute their budget to other producing factors instead of labor costs due to a higher ratio of social insurance contribution. Such a substitution effect will accelerate innovation and development, while improving business performance with lower labor cost.

For the sustainability of business operations, the increased investment in social insurance for employees could indirectly force enterprises to improve their R&D efforts and optimize the management structure, while social insurance as a kind of staff welfare, can motivate employees and improve productivity [13–15]. However, for the short-term development, social insurance contributions mean higher labor costs and a heavier operating burden for enterprises, utilizing funds that could have been invested in R&D innovation and business expansion. Labor-intensive manufacturers would be negatively affected in terms of productivity growth and expansion of production [16–18].

Social insurance premiums are recorded as corporate tax burdens. China has adopted quite a high ratio of social insurance contributions, compromising five components: pension, healthcare, work injury, maternity, and unemployment. A total rate of 40% is also at a fairly high level compared with other countries. However, the actual social insurance contribution is lower than the nominal ratio due to regional policy differences, and a huge number of companies pay the social insurance based on the basic salary instead of the actual salary for employees to save on labor costs [19–21]. According to Xu and Liu [22], the input of resources like labor, capital, and technology is what primarily drives the gross industrial output value of the manufacturing industry in China. Cai and Tian [23] found that the net asset return of enterprises with a high tax burden is significantly lower, after reducing the tax burden and social security payment burden of enterprises, the net return on assets of enterprises increased significantly. Therefore, this article proposes the following research hypotheses:

Hypothese 1: Reducing the actual social security contribution rate of enterprises is beneficial for promoting their financial performance.

Gong et al. [24] found the total number of production factors is different between SOMs and non-SOMs. The average production efficiency of SOMs is lower than that of non-SOMs. Non-SOMs have more advantages in resource allocation, which means more factors of production. Zhang et al. [25] found that the technical efficiency of non-SOMs slightly outperformed SOMs based on analysis of Data Enveloping Analysis (DEA) model and Stochastic Frontier Analysis (SFA) model. In addition, non-SOMs were more advantageous in pure technical efficiency, while SOMs perform better in scale efficiency. Yang et al. [26] proposed that lower the ratio of social security contribution, on one hand, will reduce the cost of enterprises; on the other hand, a lower ratio of contribution has a more apparent positive effect on SOMs. Therefore, this thesis proposes another research hypothese:

Hypothese 2: The reduction in the actual social security contribution rate of SOMs has a more significant promoting effect on their financial performance than non-SOMs.

In summary, the ever-changing connection between social insurance premiums and enterprise performance has been widely studied. Despite variations in the study subjects and research methodologies, the studies have arrived at a consensus that the ratio of social insurance contributions has an impact on the input of production factors in enterprises. Furthermore, national policies that involve tax cuts and fee reductions have been found to effectively reduce enterprise costs. China has implemented several policies pertaining to social insurance premiums in response to the effects of COVID-19. These policies aim to alleviate the financial burden experienced by enterprises and facilitate economic recovery. Notwithstanding the aforementioned conclusion, there is an absence of empirical research on the potential effects of a reduced ratio of social security contributions on the financial performance of both SOMs and non-SOMs. This study employs a regression model to conduct an empirical comparative analysis of the financial performance implications of a reduced contribution ratio for SOMs and non-SOMs. The study provides recommendations for the improvement and streamlining of the social insurance system.

## 3. Data model and empirical analysis

Gong et al. [24] and Zhang et al. [25] conducted empirical analyses and comparisons of the total production factors and technical efficiency of SOMs and non-SOMs using the estimation model and the DEA model, respectively. This article gathered pertinent data by referencing the variable selection and empirical methods described previously. A multiple linear regression model is created using the social insurance payment rate of SOMs and non-SOMs, corporate financial performance, and enterprise growth as explanatory and explained variables and control variables like enterprise size, board size, debt level, ratio of independent directors, equity checks and balances, enterprise age, enterprise size, and year. In order to conduct a more intuitive analysis of the potential effects of a reduction in social security payment rates on the financial performance of both state-owned and non-state-owned entities, further investigation is warranted.

### 3.1 Data sources

The data used in the empirical analysis are cited from the Guotaian CSMAR database and the WIND database. The data for all financial statements was cited from the public documents of A-share-listed SOMs and non-SOMs from 2011 to 2020. The sample collection mainly selected labor-intensive manufacturers in China, whose labor expenses make up a larger portion of operating costs, so they are more sensitive to changes in the ratio of social insurance contributions. In order to improve the real validity of the sample data, companies with suspensions or delistings, or ST companies, during 2011–2020 were excluded, so were companies with zero social insurance contributions. Based on the above conditions, this thesis selected a total of 3,691 listed companies with 15,937 observations in the non-SOMs and 483 listed companies with 3,451 observations in the SOMs.

### 3.2 Define variables

#### 3.2.1 Explanatory variables

The explanatory variable in this thesis is the ratio of social insurance contributions paid by enterprises. There are two types of explanations of the ratio of contribution: nominal and real. When the enterprise pays social insurance strictly according to the statutory standard formulated by the national policy, the ratio of social insurance contributions to total costs is called the nominal ratio. The nominal ratio is more of an ideal situation, but research shows that some enterprises do not fully pay the social insurance fees in line with the requirements of laws and regulations, so the nominal ratio is more often higher than the actual ratio. In this thesis, the “ratio of social insurance contribution” refers to the actual ratio adopted by enterprises, measured by: social insurance premiums/employee compensation payable × 100%.

#### 3.2.2. Explained variables

In this thesis, Chen and Wang [27] were referred to the impact of the reduction of pension insurance rates on the high-quality development of enterprises, and the explanatory variable was defined as the actual payment rate of social insurance enterprises. The explanatory variables in this thesis are corporate financial performance, which is the profit obtained from a company’s operations within a specified operating period. There are many indicators to measure corporate performance financially, such as the gearing ratio to reflect the solvency of a company and the sales growth rate to reflect its development capability. In this thesis, return on total assets (ROA) is used to measure financial performance because it intuitively reflects the efficiency of asset utilization and capital operation results and is an effective indicator of overall profitability and operation. The formula for calculating the ROA is as shown in Eq (1):

$$\text{Net}\;\text{profit}\;÷\;\text{Total}\;\text{average}\;\text{assets}\;\left(\text{Total}\;\text{average}\;\text{liabilities}\;\text{ten}\;\text{average}\;\text{owner}’\text{s}\;\text{equity}\right)\times 100\%=\text{ROA}$$ (1)

#### 3.2.3. Control variables

To mitigate endogeneity concerns and enhance the precision of the model, this study has incorporated control variables. The primary themes explored in the earlier research and pertinent literature have both influenced the choice of these variables. The chosen control variables encompass corporate growth (Growth), debt level (Lev), independent director ratio (Bind), equity checks and balances (CS), corporate age (AGE), corporate size (Size), board size (Bsize), and year (Year). The explanatory variable, explained variable, and control variable data utilized in this study were obtained from the CSMAR and WIND databases. Table 1 displays the specific variable indicators.

**Table 1:** Variable definition table.

Variable Type	Variable Name	Variable Symbols	Calculation of variables
Explained variables	Corporate Financial Performance	ROA	Return on Total Assets
Explanatory variables	Actual social security contribution rate	Rate	Social insurance premiums/employee compensation payable × 100%
Control variables	Business Growth	Growth	Operating income growth rate
	Debt Level	Lev	Gearing ratio
	Independent director ratio	Bind	Number of Independent Directors / Number of Directors
	Shareholding checks and balances	CS	Percentage of shareholding of the largest shareholder
	Business Age	AGE	ln (years of establishment + 1)
	Enterprise size	Size	Ln (Total assets)
	Board Size	Bsize	Number of Directors
	Year	Year	Year dummy variable

In Table 1, the financial performance of the explained variable is selected as an indicator of ROA. The actual ratio of social security contribution as the explained variable is calculated based on the ratio of social insurance premium to employee compensation payable. As for the control variable, the growth rate of business revenue is selected as an indicator. The liability is presented as an indicator of asset-liability ratio. The ratio of independent directors is calculated by dividing the number of independent directors by the number of directors. The shareholding balance is represented by the proportion of the largest shareholder. The operating years of the enterprise are the logarithm of the establishment year plus 1. The size of the enterprise is the logarithm of the total assets of the enterprise. The size of the board of directors is the number of directors, and the year is taken as the dummy variable.

### 3.3 Building the model

In this thesis, the following fixed effect regression model is constructed to further investigate the impact of a lower ratio of social security contribution on firms’ financial performance.


$$\begin{array}{l} {\text{ROA}}_{\text{it}}=\alpha_{0}+\alpha_{1}{\text{Rate}}_{\text{it}}+\alpha_{2}{\text{Growth}}_{\text{it}}+\alpha_{3}{\text{Lev}}_{\text{it}}+\alpha_{4}{\text{Bind}}_{\text{it}}+\alpha_{5}{\text{AGE}}_{\text{it}}+\alpha_{6}{\text{Size}}_{\text{it}}+\\ \alpha_{7}{\text{Bsize}}_{\text{it}}+\alpha_{8}{\text{CS}}_{\text{it}}+\Phi \text{i}+\text{Year}\;\text{t}+\varepsilon_{\text{it}} \end{array}$$ (2)

In Eq (2): i represents firm, t represents year, ROAit represents firm financial performance of firm i in year t. Φi represents individual fixed effects, Year t represents time fixed effects, and εit represents the random disturbance term.

### 3.4 Descriptive statistics and Pearson correlation test

In this thesis, descriptive statistics of selected SOM and non-SOM variables are shown in Tables 2 and 3. The explained variables are respectively selected as return on total assets, explanatory variable actual social security contribution rate, control variable operating revenue growth rate, asset-liability ratio, ratio of independent directors, shareholding ratio of the largest shareholder, enterprise age, enterprise size, number of directors and year. By comparison, it is found that the average value of the actual social security contribution rate of the non-SOMs is 9.55%, 3.57% higher than the actual social security contribution rate of the SOMs is 5.98%. At the same time, the maximum actual social security contribution rate of the non-SOMs is 98.56%, 8.68% higher than the maximum value of 89.88% in the non-SOMs. Moreover, the actual ratios of SOMs are unevenly distributed, with a large difference between the maximum and minimum values. The above situation indicates that the actual ratio for non-SOMs is generally higher than that for SOMs. Furthermore, the ROA also applies in this situation where the average ROA of SOMs is 5.03%, less than that of non-SOMs (7.74%). However, the maximum return on total assets of SOMs is 95.69%, 14.13% higher than the maximum return on assets of non-SOMs, which is 81.56%. The minimum growth rates of total return on assets and operating revenue of non-SOMs are -19.97% and -91.44%, respectively, indicating that the business performance of SOMs is better than that of non-SOMs, due to the great impact of the 2020 COVID-19 outbreak on non-SOMs whose performance plunged sharply. The mean value, maximum value and minimum value of variables such as enterprise’s age, scale, debt level, growth and number of directors of SOMs and non-SOMs were similar.

**Table 2:** Descriptive statistics of SOMs variables.

Variable	Obs	Mean	Std. dev.	Min	Max
ROA	3,198	5.029908	5.586791	0.0045	95.6893
Rate	3,198	5.975173	11.85722	0.005168	89.88216
Growth	3,198	20.65871	61.31134	0.0053	3150.222
Lev	3,198	49.17759	20.88031	1.561	229.0134
Bsize	3,198	9.061288	1.794579	4	18
Bind	3,198	0.3716731	0.0571911	0.2	0.8
CS	3,198	0.3578979	0.1402823	0.0572	0.8909
AGE	3,198	2.985901	0.25088	1.791759	3.73767
Size	3,198	22.64702	1.289344	19.07423	27.547

Source: Guotaian CSMAR database, WIND database.

**Table 3:** Descriptive statistics of non-state manufacturing industry variables.

Variable	Obs	Mean	Std. dev.	Min	Max
ROA	15,580	7.740919	7.517532	-19.9696	81.5616
Rate	15,580	9.549618	6.629369	0.2869	98.5584
Growth	14,900	15.38892	27.10657	-91.4407	196.6398
Lev	15,573	36.88483	18.16377	0.708	99.8124
Bsize	15,580	7.075417	3.129082	0	17
Bind	15,580	31.98771	14.14527	0	100
CS	12.703	34.43195	15.37537	2.87	100
AGE	15,580	2.779252	0.384331	0.6931472	4.189655
Size	15,579	21.38742	1.211332	14.48716	26.61043

Source: Guotaian CSMAR database, WIND database.

In conclusion, non-SOMs made a higher actual paid ratio of social security than SOMs did, while SOMs performed better operationally than non-SOMs did.

Tables 4 and 5 show the results of Pearson correlation test for variables. The correlation coefficients of ROA and Rate between non-SOMs and SOMs are significantly negative at the level of less than 5%, indicating that a lower actual ratio of social security contribution will improve the financial performance to some degree. The ROA of non-SOMs was significantly negatively correlated with Rate, Lev, Bsize, Bind, AGE and Size, respectively, and significantly positively correlated with Growth and CS, indicating that in addition to the promotion effect of a lower actual ratio of contribution, lower debt, larger scale, the ratio of independent directors and the size of the board of directors could all lead to an improved financial result and operating income and shareholding checks and balances. The ROA of SOMs is significantly negatively correlated with Rate, Lev, Bsize and Size, and significantly positively correlated with Growth, CS and AGE, indicating that SOMs can reduce the actual ratio, debt level, board size and enterprise size, and increase operating income, shareholding checks and balances, and enterprise age for better financial performance.

**Table 4:** Pearson correlation test of non-SOMs.

	ROA	Rate	Growth	Lev	Bsize	Bind	CS	AGE	Size
ROA	1.000								
Rate	-0.059***	1.000							
Growth	0.292***	-0.083***	1.000						
Lev	-0.259***	-0.201***	0.057***	1.000					
Bsize	-0.305***	-0.089***	0.062***	-0.111***	1.000				
Blind	-0.313***	-0.055***	0.055***	-0.157***	0.735***	1.000			
CS	0.229***	-0.086***	0.008	-0.049***	-0.174***	-0.095***	1.000		
AGE	-0.276***	-0.085***	0.149***	-0.005	0.369***	0.358***	-0.192***	1.000	
Size	-0.132***	-0.305***	0.025***	0.274***	0.462***	0.369***	-0.080***	0.360***	1.000

Note:

*** indicate coefficients significant at the 1%.

**Table 5:** Pearson correlation test of SOMs.

	ROA	Rate	Growth	Lev	Bsize	Bind	CS	AGE	Size
ROA	1.000								
Rate	-0.048***	1.000							
Growth	0.037**	-0.022	1.000						
Lev	-0.121***	0.146***	0.019	1.000					
Bsize	-0.052***	0.054***	0.038**	0.121***	1.000				
Blind	-0.011	-0.037**	0.008	0.032*	-0.327***	1.000			
CS	0.052***	0.094***	0.025	-0.001	0.002	0.066***	1.000		
AGE	0.042**	-0.220***	0.000	-0.003	0.027	0.018	-0.149***	1.000	
Size	-0.051***	-0.073***	0.042**	0.359***	0.287***	0.090***	0.188***	0.115***	1.000

Note:

***, **, and * indicate coefficients significant at the 1%, 5%, and 10% levels, respectively.

### 3.5 Analysis of fixed effect regression model results

This thesis carries out regression analysis on the relationship between the actual ratio of social security contribution and the financial performance of the SOMs and non-SOMs by citing the short panel data from 2011 to 2020. The analysis was based on the time fixed effect model with the results shown in Table 6. It can be concluded that the actual ratios of social security contribution made by SOMs and non-SOMs are significantly negatively correlated with the ROA at the 1% level. In other words, a lower ratio of contribution will improve business financial performance, which is consistent with the above correlation analysis results. The correlation coefficient between the actual ratio of contribution for non-SOMs and the ROA is -0.0107, but its absolute value is less than the absolute value of the coefficient of SOMs -0.0236, indicating that the financial performance of SOMs would be more easily impacted by a lower ratio of contribution. Reducing the ratio of contribution for SOMs by 1% would translate into an increase of 0.0237% in total return on assets. In the post-pandemic era, a fast recovery of SOMs and non-SOMs industries would be supported by a reduction in the ratio of social security contribution, particularly for SOMs who would play a more effective role in business economic recovery. According to Table 6, the operating revenue growth rate of non-SOMs, the number of directors and the shareholding ratio of substantial shareholders are significantly positively correlated with ROA, while the actual ratio of contribution, asset-liability ratio and independent director ratio are significantly negatively correlated with ROA. The operating revenue growth rate of SOMs, the shareholding ratio of the largest shareholder, and the age of the enterprise are significantly positively correlated with the financial performance of the enterprise. The actual ratio of social security contribution, asset-liability ratio, and the number of directors are significantly negatively correlated with the ROA. The long history of SOMs will facilitate their financial performance, while the operating years of non-SOMs is not significant to their financial performance.

**Table 6:** Fixed effects regression model.

Variables	Non-state-owned manufacturing enterprises	State-owned manufacturing enterprise
	ROA	ROA
Rate	-0.01070*** (-3.85)	-0.02366** (-2.27)
Growth	0.00495*** (7.40)	0.00378** (2.22)
Lev	-0.00688*** (-5.86)	-0.02749*** (-4.97)
Bind	-1.24826*** (-29.74)	-2.71547 (-1.39)
CS	0.00485*** (4.03)	2.52838*** (3.28)
AGE	-0.05190 (-0.93)	0.85148* (1.71)
Size	-0.00953 (-0.51)	-0.07882 (-0.81)
Bsize	0.05571** (2.38)	-0.13836** (-2.09)
Constant	52.17380*** (32.23)	7.66019*** (3.12)
Observations	12,660	3,190
Number of code	1,472	346
R-squared	0.087	0.032
F-test	62.50	5.486

t-statistics in parentheses;

*** *P* <0.01,

** *P* < 0.05,

* *P* < 0.1.

In conclusion, both SOMs and non-SOMs can improve their own financial performance by reducing the actual ratio of social security contribution and asset-liability ratio, increasing the growth rate of operating revenue and the shareholding proportion of the largest shareholder in the post-pandemic era, leading to a faster recovery of economy and business, moreover, the effect of reducing the social security contribution rate of SOMs on promoting financial performance is more significant than that of non-SOMs. Therefore, the previous research hypothese 1 and research hypothese 2 are valid.

### 3.6 Analysis of variance

In order to test the overall significance of the fixed effect model, the variance analysis method is employed. In order to achieve the purpose of assessing whether the influence of explanatory variables on explained variables is significant, it is necessary to test the significance of the fixed effects model, which is generally measured by F value and P value. In this thesis, the overall significance test of the two models for non-SOMs and SOMs is shown in Tables 7 and 8.

**Table 7:** Analysis of variance of non-SOMs.

Source	Partial SS	df	MS	F	Prob>F
Model	156192.43	8	19524.054	630.41	0.0000
Rate	13806.605	1	13806.605	445.80	0.0000
Growth	40336.464	1	40336.464	1302.42	0.0000
Lev	59525.363	1	59525.363	1922.00	0.0000
Bind	6704.0743	1	6704.0743	216.47	0.0000
CS	13762.388	1	13762.388	444.37	0.0000
AGE	2174.9022	1	2174.9022	70.23	0.0000
Size	134.32049	1	134.32049	4.34	0.0373
Bsize	622.4299	1	622.4299	20.10	0.0000
Residual	391807.51	12,651	30.970478		
Total	547999.94	12,659	43.289355		

**Table 8:** Analysis of variance of SOMs.

Source	Partial SS	df	MS	F	Prob>F
Model	2438.2754	8	304.78443	9.97	0.0000
Rate	66.285123	1	66.285123	2.17	0.0000
Growth	143.04304	1	143.04304	4.68	0.0306
Lev	929.68448	1	929.68448	30.40	0.0000
Bind	57.919227	1	57.919227	1.89	0.1689
CS	421.73365	1	421.73365	13.79	0.0002
AGE	220.42484	1	220.932366	7.21	0.0073
Size	20.932366	1	20.932366	0.68	0.4081
Bsize	132.63281	1	132.63281	4.34	0.0374
Residual	97281.355	3,181	30.582004		
Total	99719.631	3,189	31.269875		

According to Table 7, the F-test value of the model is 630.41, and the P value of the significance test of the model is 0.000 (*P*<0.005), indicating that the F-test is significant. The model was set reasonably through the test, and the explanatory variables could well explain the explained variables. The F-tests for the other variables Rate, Growth, Lev, Bind, CS, AGE, Size and Bsize are also all positively significant at the 1% level, indicating that each variable has an impact on the differences in ROA.

According to Table 8, the F test value of the model is 9.97, and the P value of the significance test of the model is 0.000 (*P*<0.005), indicating that the F test was significant. Through the test, the model was set reasonably, and the explanatory variables could well explain the explained variables. The F-tests for the other variables Rate, Growth, Lev, CS, AGE and Bsize are also all positively significant at the 5% level, indicating that each variable has an impact on the differences in ROA.

### 3.7 VIF test

The variance inflation factor (VIF) evaluates the model’s multicollinearity problem. The VIF test’s results of non-SOMs and SOMs are shown in Table 9. The multicollinearity problem becomes more severe as VIF size increases. When the maximal VIF is less than 10, it indicates that there is no multicollinearity issue with the model. The mean values of the co-linearity test in the fixed effect models of SOMs and non-SOMs are 1.17 and 1.15, respectively, which are both less than 5, and the maximum VIF is 1.42, indicating that there is no multicollinearity issue between the variables in the fixed effect models of SOMs and non-SOMs.

**Table 9:** VIF test of non-SOMs and SOMs.

	State-owned manufacturing industry		Non-state-owned manufacturing industry	
Variable	VIF	1/VIF	VIF	1/VIF
Size	1.39	0.717157	1.42	0.705686
Bsize	1.27	0.785708	1.09	0.916681
Lev	1.20	0.831792	1.26	0.796641
Bind	1.17	0.855212	1.03	0.972628
Rate	1.11	0.904310	1.14	0.876343
CS	1.09	0.915770	1.08	0.926483
AGE	1.09	0.918182	1.12	0.891648
Growth	1.00	0.995071	1.04	0.965809
Mean VIF	1.17		1.15	

### 3.8 Robustness test

To test the robustness of the results of the empirical analysis, this thesis replaces the explanatory variables to test the results of the previous regression analysis. Since the ROE and ROA are both important indicators in the measurement of business financial performance, the regression analysis is conducted after replacing the original explanatory variable, corporate performance (ROA), with return on net assets (ROE) in the robustness test. The regression results upon the variable replacement are shown in Table 10. With explanatory variables replaced, the ratio of social security contribution and ROA of SOMs and non-SOMs are significantly negatively correlated, indicating that the regression results after the variable replacement are consistent with the baseline regression results, and the baseline regression results are valid through the robustness test. In other words, a lower actual ratio of social security contributions will improve the financial performance.

**Table 10:** Analysis of multiple linear regression results after substitution variables.

Variables	State-owned manufacturing	Non-State Manufacturing
	ROE	ROE
Rate	-0.0481*** (-4.78)	-0.218*** (-5.52)
Size	0.2105*** (11.86)	0.046*** (-10.62)
Lev	-0.2862*** (27.65)	-0.400*** (-18.89)
Growth	0.02632*** (8.75)	0.088*** (-23.03)
AGE	0.0356* (3.25)	0.101*** (-4.96)
Bsize	-0.0004 (0.32)	-0.001 (-0.33)
Bind	-0.0762** (-2.12)	-0.027 (-0.56)
CS	0.0934*** (7.18)	0.002*** (-6.07)
_cons	0.0456*** (-8.58)	-1.020*** (-9.54)
Individual companies	Control	Control
Year	Control	Control
r2	0.2481	0.231
N	3198	15580

Note:

***, **, and * indicate coefficients significant at the 1%, 5%, and 10% levels, respectively.

## 4. Conclusions and recommendations

### 4.1 Conclusions

This thesis analyzes the relationship between the actual ratio of social security contribution and the financial performance of SOMs and non-SOMs by establishing a fixed-effects regression model. It is found that the actual ratio of social security contribution and the total average ROA in SOMs are higher than those of non-SOMs. When the actual ratio of contribution is equal, the gap between the maximum and minimal ROA in SOMs is huge and unevenly distributed. Some S0OMs have better management, so the actual ratio of contribution is higher, while some non-SOMs poor operation leads to a lower actual ratio of contribution. In summary, the ratios of contribution adopted by SOMs and non-SOMs are polarized. In addition, the actual ratio of contribution and the ROA were significantly negatively correlated in both SOMs and non-SOMs. To be specific, a lower ratio of contribution can improve an enterprise’s financial performance, but the absolute value of the correlation coefficient between the actual ratio of contribution and ROA in non-SOMs is greater than in SOMs. Indeed, a lower ratio of contributions for SOMs would boost their financial performance.

### 4.2 Recommendations

(1) Implement tax and premium reduction policies. The general ratio of social insurance contributions implemented in China is higher than the international average level, which could translate into a heavy burden shouldered by enterprises. Pension insurance premiums account for the largest part of the social security package. Facing the dilemma of an increasing aging population and high pension insurance premium rates, the government should reduce the burden for enterprises based on the actual situation while balancing a lower ratio of pension contribution with guaranteed pension payment mechanisms by effectively coordinating with enterprises. In addition, when formulating specific social insurance policies, local governments should take the particular operating conditions of different companies into full consideration, managing to find the balance between social welfare and financial performance. Many enterprises suffered financial crises due to the outbreak of COVID-19, and social insurance contributions have become a heavy burden for enterprises that are losing growth momentum. To motivate business development, the Chinese government should try to find solutions with a lower ratio of social insurance contributions. According to the above empirical study, a lower ratio of social security could play a more positive role in non-SOMs’ financial performance. That is to say, how to reduce the actual ratio of social security contributions for SOMs should be the new focus for the Chinese government.

(2) Implement a differentiated social insurance contribution policy. The ratio of social insurance contributions applied to enterprises in different industries varies. Therefore, local governments should also take into account the differences among enterprises when determining the ratio of contribution and formulating relevant policies accordingly. It is important to ensure that enterprises are subject to a fair and just ratio of contribution that adapts to individual conditions. At the same time, regions with better conditions may promote commercial insurance as a supplement to the social security system.

(3) Strengthen the supervision of social insurance contributions. At present, the actual ratio of social insurance contributions is lower than the nominal ratio, and some enterprises do not pay social insurance in full and on time as required. Those who violate relevant regulations and policies bear lower labor costs than compliant ones, which will translate into unfair market competition, hurting the sustainability of a healthy business environment. To build a level playing field, the government should leverage the measures of tax and premium reduction to maintain a fair and just market with enhanced management, thus stimulating the enthusiasm of enterprises to contribute to the social insurance fund. In this way, we could maintain a healthy and sustainable operation of the social insurance system.

## Supporting information

S1 File (ZIP)

## Abbreviations

AGE: Corporate Age
Bind: Independent Director Ratio
Bsize: Board Size
CS: Equity Checks and Balances
CSMAR: Database China Stock Market & Accounting Research Database
DEA model: Data Enveloping Analysis model
i: Firm
Lev: Debt Level
Ln: Total Assets
ln: Years of Establishment + 1
R&D: Research and Development
ROA: Return on Total Assets
ROAit: Firm financial performance of firm i in year t
ROE: Return on Net Assets
SFA model: Stochastic Frontier Analysis
SOE: State-owned Enterprise
SOMs: State-owned Manufacturers
t: Year
VIF: Variance Inflation Factor
Year t: Time Fixed Effects
εit: The Random Disturbance Term
Φi: Individual Fixed Effects
