# Supplementary material for: Reducing social security contribution rate on the financial performance of state-owned and non-state-owned manufacturing industries in the post-epidemic era
Source: PLoS One. 2023 Jun 23;18(6):e0287596. doi: 10.1371/journal.pone.0287596 (PMC10289345; doi:10.1371/journal.pone.0287596)
Supplement: S1 File — (ZIP) [file pone.0287596.s001.zip › Steps of empirical analysis.doc]

**1.Descriptive statistics of state-owned manufacturing and non-state-owned manufacturing variables**

Table1 Descriptive statistics of state-owned manufacturing variables

Data sources: Guotai 'an CSMAR database, WIND database

Table2 Descriptive statistics of non-state-owned manufacturing variables

Data sources: Guotai 'an CSMAR database, WIND database

**2.Correlation analysis of variables of state-owned and non-state-owned manufacturing industries**

Table3 Pearson correlation test of non-state-owned enterprises

Table 4 Pearson correlation test of state-owned enterprises

**3Fixed effect model analysis of state-owned manufacturing and non-state-owned manufacturing**

Table5 Fixed effect model of state-owned manufacturing industry and non-state-owned manufacturing industry

| VARIABLES | Non-state-owned manufacturing industry | State-owned manufacturing industry |
| --- | --- | --- |
|  | ROA | ROA |
| Rate | -0.01070***  (-3.85) | -0.02366**  (-2.27) |
| Growth | 0.00495***  (7.40) | 0.00378**  (2.22) |
| Lev | -0.00688***  (-5.86) | -0.02749***  (-4.97) |
| Bind | -1.24826***  (-29.74) | -2.71547  (-1.39) |
| CS | 0.00485***  (4.03) | 2.52838***  (3.28) |
| AGE | -0.05190  (-0.93) | 0.85148*  (1.71) |
| Size | -0.00953  (-0.51) | -0.07882  (-0.81) |
| Bsize | 0.05571**  (2.38) | -0.13836**  (-2.09) |
| Constant | 52.17380***  (32.23) | 7.66019***  (3.12) |
| Observations | 12,660 | 3,190 |
| Number of code | 1,472 | 346 |
| R-squared | 0.087 | 0.032 |
| F-test | 62.50 | 5.486 |

t-statistics in parentheses

*** p<0.01, ** p<0.05, * p<0.1

**4.Variance analysis of state-owned and non-state-owned manufacturing industries**

**Table 6** Analysis of variance of non-state-owned manufacturing industry

**Table7** Analysis of variance of state-owned manufacturing industry

**5.VIF collinearity test of state-owned manufacturing industry and non-state-owned manufacturing industry**

Table 8	VIF test of state-owned manufacturing industry and non-state-owned manufacturing industry

**6.Robustness test**

**Table 9 Analysis of fixed effects results after substitution variables**

| variables | State-owned manufacturing industry | Non-state-owned manufacturing industry |
| --- | --- | --- |
|  | ROE | ROE |
| Rate | -0.0481^***^  (-4.78) | -0.218^***^  (-5.52) |
| Size | 0.2105^***^  (11.86) | 0.046^***^  (-10.62) |
| Lev | -0.2862^***^  (27.65) | -0.400^***^  (-18.89) |
| Growth | 0.02632^***^  (8.75) | 0.088^***^  (-23.03) |
| AGE | 0.0356^*^  (3.25) | 0.101^***^  (-4.96) |
| Bsize | -0.0004  (0.32) | -0.001  (-0.33) |
| Bind | -0.0762^**^  (-2.12) | -0.027  (-0.56) |
| CS | 0.0934^***^  (7.18) | 0.002^***^  (-6.07) |
| _cons | 0.0456^***^  (-8.58) | -1.020^***^  (-9.54) |
| Enterprise individual | control | control |
| Year | control | control |
| r2 | 0.2481 | 0.231 |
| N | 3198 | 15580 |

Note: ***, **, and * indicate that the coefficients are significant at 1%, 5%, and 10% levels, respectively.
